# The value of simple microbiological studies for on-site screening of acute neonatal conjunctivitis in Angola

**DOI:** 10.1186/1869-5760-4-1

**Published:** 2014-01-25

**Authors:** Isabel Alexandre, Nestor Cortes, Mar Justel, Itziar Fernández, Raul Ortíz de Lejarazu, J Carlos Pastor

**Affiliations:** 1 Instituto Oftalmológico Nacional de Angola, Ministerio de Saúde, Luanda 2177, Angola; 2 Departamento de Oftalmología, Faculdade de Medicina, Universidade Agostinho Neto, Luanda 2177, Angola; 3 Department of Ophthalmology, Hospital Clinico Universitario, Valladolid 47005, Spain; 4 Department of Microbiology and Immunology, Hospital Clinico Universitario, Valladolid 47005, Spain; 5 Instituto de Oftalmobiología Aplicada (IOBA), University of Valladolid, Campus Miguel Delibes, Paseo de Belen 17, Valladolid 47011, Spain; 6 CIBER-BBN, Instituto de Salud Carlos III, Madrid 28029, Spain

**Keywords:** Conjunctival smears, Gram stain, Methylene blue stain, Ophthalmia neonatorum screening, Angola

## Abstract

**Background:**

Neonatal conjunctivitis or ophthalmia neonatorum (ON) is an acute bacterial conjunctivitis contracted by newborns during delivery. In non-industrialized countries, detection of the etiological agent is difficult due to the unavailability of modern diagnostic resources. Therefore, we analyzed the effectiveness of Gram and methylene blue staining techniques, which are simple microbiological methods in suspecting the aetiology of ON in a maternity ward in Luanda, Angola.

**Findings:**

Neonatal conjunctival smears (*n* = 95), maternal data, and perinatal factors were collected. Slides were air-dried and sent to the Microbiology Department of the Hospital Clinico Universitario, Valladolid, Spain, where trained personnel performed Gram and methylene blue staining methods. Findings were interpreted by two expert microbiologists. Ophthalmological examination of all children showed five newborns with clinical signs of ON. Fourteen mothers reported were suspected with vulvo-vaginitis, and 27 had a urinary infection during pregnancy. Gram staining revealed the presence of epithelial cells in 87.6% and leukocytes in 15% of the conjunctival smears. These values were significantly higher than those shown by methylene blue staining. No rods, cocci, or yeasts were identified by either staining method. *Chlamydia trachomatis* DNA was also undetected in a small sub-sample with clinical suspicion of ON. There was no correlation among the presence of ON, ON microbes, maternal data, or perinatal factors.

**Conclusions:**

Basic microbiological techniques did not provide enough information for screening cases of ON in Angola. Therefore, the use of molecular biology or other techniques is warranted for this purpose.

## Findings

Neonatal conjunctivitis, or ophthalmia neonatorum (ON), has been a major health problem for many centuries [1]. ON develops during the first month of life, and it was originally referred as conjunctivitis caused by *Neisseria gonorrhoeae*. Now this term refers to any acute conjunctivitis (AC) developed in this age group, irrespective of the causative agent [2]. ON produced by sexually transmitted pathogens have declined as a result of the decreased prevalence of these infections as well as routine prenatal screenings [3]. ON occurred in 1% to 12% of newborns and it is a mild disease with the exception of *N. gonorrhoeae*[3]. Without prevention, ON occurs in 30% to 42% of infants exposed to *N. gonorrhoeae* and may progress to corneal ulceration [4]. Today, blindness from ON is rare in industrialized countries [1], although it is still a problem in many areas of developing countries [5].

In Angola, after 30 years of civil war, the health care system has serious deficiencies including the lack of systematic prophylaxis for ON. In 2009, Pastor and Alexandre reported that in one maternity ward in Luanda, Angola, 12.3% of the children presented with clinically diagnosed bilateral AC [6]. Microbiological examinations were not available and there was no information on the causative agents. Today, agents for ON include *Chlamydia trachomatis* (CT), *Staphylococcus*, *Streptococcus*, *Haemophilus*, and others in addition to *N. gonorrhoea*[2,3]. Thus, effective treatment requires determining the causative agent.

In 2009, we started in Angola a project with the aim of developing a national programme for ON prophylaxis. Because access to microbiology techniques is complicated, we tried to provide the ophthalmologists the simplest tools to screen the pathogenesis of AC. Two simple and inexpensive staining techniques (ST), Gram and methylene blue (MB), were selected. Although they are unable to detect CT, they provide basic information about other potential ON agents. Thus, the purpose of this study was to analyze the clinical usefulness of the Gram and MB on conjunctival smears collected from newborns in a maternity ward in Luanda.

### Patients, materials, and methods

The project was approved by Ethical Commission (Agostinho Neto University, Luanda). It was a prospective, non-interventional study including healthy newborns delivered at the Augusto N’ Gangula Hospital from March to June 2010. It was conducted according to the Declaration of Helsinki.

Inclusion criteria were limited to those newborns having a gestational time of 37 to 40 weeks and birth weight of ≥ 2.300 kg whose mothers signed the informed consent.

Neonatal weight, Apgar score, delivery method, mother's age, ethnicity, residency, parity, prenatal care, and suspected or confirmed vaginal or urinary infections during pregnancy were recorded.

Samples were taken by the ophthalmologist, immediately after delivery, without using eyelid speculum and topical anaesthetics, with sterile swabs from the lower palpebral-conjunctival surface of both eyes. Each sample was smeared onto glass slides. Each slide was air-dried, fixed, and placed at room temperature. The slides were stored for a maximum of 3 weeks before shipping to the Department of Microbiology, Hospital Clinico Universitario, Valladolid, Spain, where they were stained with Gram and MB and analyzed by two microbiologists who examined at least 20 microscope fields per stain at × 1,000 and agree on the final result.

The presence of epithelial cells (EC), leukocytes, rods, cocci, and yeasts was assessed and rated per five microscope fields. Geno Quick® technique (Hain Lifescience, Nerhen, Germany) was used for CT*-*DNA in ten slides from newborns with clinical signs of AC (discharge, redness, conjunctiva, and eyelid swelling).

After taking the samples, all newborns were given an examination including pupil and light responses, eyelid, conjunctiva, cornea, iris, and lens transparency, and retinal red reflex (Brückner test).

### Statistical analyses

Normality was checked (Lilliefords' approximation to Kolmogorov-Smirnov test). Weighted kappa coefficient was used to evaluate the agreement between eyes and ST. Differences between eyes and ST were analyzed by the equality of two-proportions test. The relationship between microbiological and neonatal-maternal characteristics was evaluated by Spearman's correlation coefficient and Kruskal-Wallis analysis of variance. Statistical analyses were performed using R software [7] and package vcd [8]. The *p* values ≤ 0.05 were considered as significant.

## Results

The data from 95 newborns and mothers were analyzed. Mother's age was 24.39 ± 6.29 years (range 15 to 42). Most of them, 55.7%, came from low socioeconomic neighbourhoods. Prenatal visits were 5.14 ± 2.5 (range 0 to 9), but 14 mothers had no medical attention before labour. Mean parity was 1.74 ± 1.78. Most births were vaginal (84.2%) and 15 cases were by caesarean delivery (15.7%). Fourteen mothers (14.7%) reported suspected or confirmed vulvo-vaginitis during pregnancy and 27 (28.4%) with urinary infection. Forty-eight newborns were male (54.6%) and 40 (45.5%) were females. In the seven cases, the sex of the newborns was not noted.

All newborns had normal pupil reflexes and Brückner test. Eleven (11.6%) had pathologic findings and five with signs of AC.

All Geno Quick® results were negative. Rods, cocci, and yeast cells were absent in all slides. Cases without EC (36.8%) were significantly higher with MB (*p* = 0.0002, Table 1). The percentage with at least ten EC was significantly higher with Gram (*p* < 0.0001, Table 1). There were no significant differences in the other EC groups. Leukocytes were rarely seen by either ST (Table 1), and there were no significant differences by any method. There was low to moderate agreement on the presence of EC and leukocytes between eyes and between ST (Table 2). Because no microbes were detected, there were no correlations with neonatal-maternal characteristics.

**Table 1:** Presence of epithelial cells and leukocytes in the cytological examination

**Cell presence**			**Methylene blue stain**				**Gram stain**				***p *** value*
			***N***	**%**	**95% CI (%)**		***N***	**%**	**95% CI (%)**		
					**Lower limit**	**Upper limit**			**Lower limit**	**Upper limit**	
Epithelial cells	LE	None	35	36.8	27.35	47.41	12	12.6	6.98	21.41	0.0002
		≤ 5	41	43.2	33.16	53.71	43	45.3	35.14	55.78	0.8839
		6-9	14	14.7	8.58	23.83	12	12.6	6.98	21.41	0.8328
		≥ 10	5	5.3	1.95	12.42	28	29.5	20.79	39.84	< 0.0001
	RE	None	35	36.8	27.35	47.41	11	11.6	6.2	20.18	0.0001
		≤ 5	39	41.1	31.21	51.63	28	29.5	20.79	39.84	0.1289
		6-9	15	15.8	9.4	25.03	27	28.4	19.87	38.74	0.0545
		≥ 10	6	6.3	2.59	13.77	29	30.5	21.71	40.94	< 0.0001
Leukocytes	LE	None	88	92.6	84.91	96.73	81	85.3	76.17	91.42	0.1651
		≤ 5	5	5.3	1.95	12.42	11	11.6	6.2	20.18	0.1915
		6-9	2	2.1	0.37	8.13	3	3.2	0.82	9.61	1.0000
		≥ 10	0	0	0	4.84	0	0	0	4.84	
	RE	None	92	96.8	90.39	99.18	87	91.6	83.61	96.03	0.2140
		≤ 5	2	2.1	0.37	8.13	3	3.2	0.82	9.61	1.0000
		6-9	1	1.1	0.05	6.56	2	2.1	0.37	8.13	1.0000
		≥ 10	0	0	0	4.84	3	3.2	0.82	9.61	0.2445

RE right eye; LE left eye; *Equality proportions test.

**Table 2:** Agreement between eyes and staining techniques

			**Concordance rate**				**Weighted kappa coefficient**		
			***N***	**%**	**95% CI of concordance rate**		**Kappa**	**95% CI of kappa**	
					**Lower limit**	**Upper limit**		**Lower limit**	**Upper limit**
Methylene blue stain vs. Gram stain	Epithelial cells	LE	33	34.7	25.5	45.3	0.128	−0.0961	0.3516
		RE	38	40.0	30.2	50.6	0.243	−0.0029	0.4882
	Leukocytes	LE	83	87.4	78.6	93.0	0.412	0.3074	0.5167
		RE	89	93.7	86.2	97.4	0.381	0.3097	0.4525
RE vs. LE	Epithelial cells	Methylene blue stain	61	64.2	53.7	73.7	0.524	0.2990	0.7495
		Gram stain	36	37.9	28.3	48.5	0.174	−0.0543	0.4024
	Leukocytes	Methylene blue stain	88	92.6	85	96.7	0.281	0.2104	0.3514
		Gram stain	80	84.2	75	90.6	0.373	0.2673	0.4796

RE right eye; LE left eye. Concordance values can vary between 0 (no concordance) and 1 (perfect concordance). Moderate concordance was considered to be 0.4 to 0.5, and good concordance was considered to be ≥ 0.6.

## Discussion

This is the first time that simple and affordable microbiological methods for ON screening have been assayed in a population of newborns in Luanda. This work has some limitations derived mainly from the lack of experience of the local ophthalmologists in performing clinical studies and collecting data in an appropriate form, but it can be considered as the first attempt to develop ophthalmic clinical research in Angola. Results are not tried to be generalized but to contribute to convince the health authorities to adopt some prophylactic measures to reduce the burden of ON.

Culture and real time-PCR techniques are currently considered the most accurate, but routine use of these technologies in Angola is not yet possible and the goal of this work was to determine if was possible to provide the on-site ophthalmologists with some easy-to-perform screening tools that are able to reveal the possible aetiology of ON. Gram and MB staining techniques were considered as appropriate options [9] to exclude most of the ON microorganisms. Although Giemsa is the first method used for CT infection, MB and Gram stains are technically less complicated and Giemsa lacks sensitivity in detecting sexually transmitted infections [10].

Gram and MB stainings are starting techniques for almost any microbiological study, although not all bacteria can be definitively classified due to the existence of Gram-variable and Gram-indeterminate groups. These include CT which is neither stained by Gram nor MB. Nevertheless, Gram stain has proven to be as effective as PCR, particularly with regards to gonorrhoea, at least for urethritis [11]. Negative results found in this study are probably derived from the different bio-burden of these two diseases.

MB, one of the simplest ST, lacks on differentiation among bacteria but helps microbiologists discern among rod-, spherical-, and spiral-shaped bacteria [12]. Results for both techniques, in this study, have been discouraging.

The presence of EC was used for comparing efficacy of both ST and to rule out the existence of technical problems when smears were taken [13]. Conjunctival leukocytes can be interpreted as an indirect sign of bacterial conjunctivitis, although they can be present in normal eyes [13,14]. In our series 10% of cases showed leukocytes, although there was no clear relationship with clinical signs. Five cases showed clinical signs of AC, and only one case had leukocytes.

Conjunctival scraping is useful for AC diagnosis in children [15,16] but it was not considered in this study because of its aggressiveness for the ocular surface. Nevertheless, it is possible that scraping was more efficient.

There was no correlation between clinical cases of AC and clinical parameters from the mother, although they are considered as risk factors [17]. Although the scarce number of samples was analyzed by PCR, no cases of CT were identified. While the incidence of ON due to CT in Angola is not known, the prevalence in Africa of ON due to *N. gonorrhoea* and CT is 50 per 1,000 newborns [18-20]. Angola could be one of the countries with lowest prevalence of CT and it may be necessary to develop culture techniques suitable for Angolan environment for some of the causative agents of ON, although CT is a fastidious microorganism who needs sophisticated cell culture medium.

There are few recent papers on this topic. In one, from Togo [21], the authors found that 8% of AC cases are more frequent in newborns delivered via vagina and mostly associated to sexually transmitted infections. *Staphylococcus aureus* was present in 25% of cases. Authors suggested that educational efforts should be made in neonatal centres. The other was made in India [22], where the prevalence of ON was low (2,6%), although authors refer that rates varies from 0.5% to 33%. The commonest organism was coagulase-negative *Staphylococcus.*

In summary, although it is feasible for ophthalmologists to perform Gram and MB staining techniques on conjunctival smears, they do not provide etiological information; thus; the use of more sophisticated techniques is warranted.

## Abbreviations

AC: Acute conjunctivitis; CT: *Chlamydia trachomatis*; EC: Epithelial cells; MB: Methylene blue stain; ON: Ophthalmia neonatorum; PCR: Polymerase chain reaction; ST: Staining techniques: Gram and methylene blue.

## Competing interests

The authors declare that they have no competing interests.

## Authors' contributions

IA carried out the preparation in Luanda including obtaining the ethical approval, organized the ophthalmologist team in Luanda, examined the newborns, took the smear samples, and drafted the manuscript. NC contributed in training the involved personnel in Luanda, took the samples, and revised the manuscript. MJ made the microbiological examinations and drafted the manuscript. IF participated in the design of the study and performed the statistical analysis. ROL made the microbiological examinations, organized the technicians in performing the staining, and drafted the manuscript. JCP conceived of the study, participated in its design and coordination, and helped draft the manuscript. All authors read and approved the final manuscript.

## Authors' information

IA is an ophthalmologist and professor of Ophthalmology department at the Agostinho Neto University Medical School, Luanda, Angola. She is also the academy director of the Instituto Oftalmologico Nacional de Angola, Luanda, Angola.NC is an ophthalmologist. During the project he was a resident in Ophthalmology department at the Hospital Clinico Universitario, Valladolid, Spain. MJ is a resident in Microbiology department at the Hospital Clinico Universitario, Valladolid, Spain. IF is a statistician and member of the CIBER-BBN, Instituto de Salud Carlos III, Madrid, Spain and the IOBA (Eye Institute) University of Valladolid, Valladolid, Spain. RO is a microbiologist and the head of the Microbiology Department at the Hospital Clinico Universitario, Valladolid, Spain.JCP is a full professor of Ophthalmology and the chairman at the Hospital Universitario de Valladolid, Spain. He is also the director of the IOBA (Eye Institute), University of Valladolid, Valladolid, Spain.
